# Early adaptive immune suppression in children with septic shock: a prospective observational study

**DOI:** 10.1186/cc13980

**Published:** 2014-07-08

**Authors:** Jennifer A Muszynski, Ryan Nofziger, Kristin Greathouse, Lisa Steele, Lisa Hanson-Huber, Jyotsna Nateri, Mark W Hall

**Affiliations:** 1 Division of Critical Care Medicine, Nationwide Children’s Hospital, 700 Children’s Drive, Columbus, OH 43205, USA; 2 The Research Institute at Nationwide Children’s Hospital, Columbus, OH 43205, USA; 3 Division of Critical Care Medicine, Akron Children’s Hospital, Akron, OH 44325, USA

## Abstract

**Introduction:**

Innate immune suppression occurs commonly in pediatric critical illness, in which it is associated with adverse outcomes. Less is known about the adaptive immune response in critically ill children with sepsis. We designed a single-center prospective, observational study to test the hypothesis that children with septic shock would have decreased adaptive immune function compared with healthy children and that among children with sepsis, lower adaptive immune function would be associated with the development of persistent infection or new nosocomial infection.

**Methods:**

Children (18 years or younger) who were admitted to the pediatric intensive care unit with septic shock (by International Consensus Criteria) were enrolled in the study. Blood samples were taken within 48 hours of sepsis onset and again on Day 7 of illness. Adaptive immune function was assessed with *ex vivo* phytohemagglutinin (PHA)-induced cytokine production capacity of isolated CD4^+^ T cells. Percentage of regulatory T cells was measured with flow cytometry. Absolute lymphocyte counts were recorded when available.

**Results:**

In total, 22 children with septic shock and eight healthy controls were enrolled. Compared with those from healthy children, CD4^+^ T cells isolated from septic shock children on Days 1 to 2 of illness and stimulated with PHA produced less of the pro-inflammatory cytokine interferon gamma (IFN-γ) (*P* = 0.002), and the antiinflammatory cytokines interleukin (IL)-4 (*P* = 0.03) and IL-10 (*P* = 0.02). Among septic shock children, those who went on to develop persistent or nosocomial infection had decreased T-cell *ex vivo* PHA-induced production of IFN-γ (*P* = 0.01), IL-2 (*P* = 0.01), IL-4 (*P* = 0.008), and IL-10 (*P* = 0.001) compared with septic shock children who did not. Percentage of regulatory T cells (CD4^+^CD25^+^CD127^lo^) did not differ among groups.

**Conclusions:**

Adaptive immune suppression may occur early in the course of pediatric septic shock and is associated with adverse infection-related outcomes.

## Introduction

Despite improvements in critical care, sepsis continues to be an important source of morbidity and mortality in children [1,2]. Successful recovery from sepsis requires resolution of initial infection and prevention of new nosocomial infections. Whereas an intact immune response is required to combat infection, critically ill adults and children commonly demonstrate immune suppression. In critically ill children, innate immune suppression (for example, monocyte function) has been described across a broad range of diagnoses, including sepsis, where it is associated with increased risks of mortality and nosocomial infection [3-6]. Previous studies suggest that both innate and adaptive immune suppression may commonly occur in adults with sepsis, associated with poor outcomes [7-11].

CD4^+^ T-helper cells are an integral cell type in the adaptive immune response. Adult studies demonstrate alterations in CD4^+^ T cells in septic shock patients compared with nonseptic controls, including increased lymphocyte apoptosis; decreased proliferative capacity in response to antigen; skewing toward T cells that promote antiinflammatory cytokine responses (for example, TH_2_); and increased percentage of immunosuppressive regulatory T cells (Treg) [9,12-15]. Further, these antiinflammatory T-cell phenotypes in adults are associated with increased risks of sepsis-related mortality [10,15]. Gene-expression studies in both adults and children likewise suggest downregulation of adaptive immune responses in sepsis [16-18]. Functional analyses of the adaptive immune response in septic shock children are lacking, and relationships between adaptive immune function and outcomes are unknown in this population.

We designed a prospective, observational study to test the hypothesis that suppressed adaptive immune function, measured with *ex vivo* CD4^+^ T-cell cytokine production capacity, would be associated with the development of persistent or nosocomial infection in children with septic shock. We further hypothesized that septic shock children would have increased percentage of Treg among CD4^+^ T cells compared with healthy children, and that among septic shock children, an increased percentage of Treg would be associated with the development of persistent or nosocomial infection.

## Methods

### Setting

The pediatric intensive care unit (PICU) at Nationwide Children’s Hospital is a 40-bed, mixed medical-surgical, quaternary care unit with more than 2,000 admissions annually. Patients admitted to a separate cardiothoracic intensive care unit were not included in this study. The study was approved by the institutional review board at Nationwide Children’s Hospital. Informed consent provided by the parent or guardian and, where appropriate, assent provided by the patient were obtained for all subjects before study enrollment.

### Subjects

Patients younger than 18 years were prospectively enrolled after admission to the PICU at Nationwide Children’s Hospital with a diagnosis of septic shock, as defined by International Consensus Criteria [19]. Subjects were excluded if they had a weight of less than 3.5 kg, a white blood cell count less than 1,000 cells/mm^3^, a limitation-of-care order in place, or a high likelihood of progression to brain death (as determined by the primary treatment team). These exclusion criteria were chosen to enroll patients for whom blood samples could be safely obtained, who would have leukocytes available for study, and who would likely survive for the duration of a 7-day sampling window. Healthy control subjects were enrolled from the outpatient phlebotomy area. Healthy subjects were excluded if they had fever, a history of chronic inflammatory disease, transplant or malignancy, or were currently taking treatment doses of antibiotics or immunosuppressive medications (including systemic corticosteroids within the past month or nonsteroidal antiinflammatory medications within the past 48 hours).

### Patient sampling

For sepsis patients, blood samples were collected within 48 hours of the onset of septic shock and again on Day 7 of illness. Day 7 samples were not obtained from patients who were no longer in the hospital or who no longer had an indwelling vascular catheter for painless blood draws. Blood samples were collected once from healthy control subjects. Samples for cell isolation and flow cytometry were drawn in EDTA tubes. Samples for whole-blood endotoxin stimulation studies and plasma cytokines were drawn in heparin tubes (Becton Dickinson). All samples were kept on ice and processed within 30 minutes of collection. Absolute lymphocyte counts were calculated from complete blood cell count with differential tests performed as part of routine clinical care.

### CD4^+^ T-cell isolation and adaptive immune function

To evaluate adaptive immune function, CD4+ T cells were isolated and stimulated as follows. Peripheral blood mononuclear cells were collected with density gradient centrifugation by using lymphocyte-separation medium (Mediatech, Manassas, VA, USA). CD4^+^ T cells were then isolated with negative selection by using magnetic beads (Miltenyi Biotec, Auburn, CA, USA). T cells, 5 × 10^5^ CD4^+^, were incubated for 24 hours at 37°C in complete media (RPMI + 10% fetal bovine serum + 1% penicillin/streptomycin) in the presence or absence of 10 μg/ml of phytohemagglutinin (PHA) (Sigma*,* St. Louis, MO, USA). After 24 hours, the supernatant was collected and stored at −80°C for subsequent cytokine analysis. Production of the cytokines IL-2, IL-4, IL-10, and IFN-γ from stimulated cells was determined with multiplex immunoassay (Bio-Rad, Hercules, CA, USA).

Percentage of regulatory T cells (CD4^+^, CD25^+^, CD127^lo^) among freshly isolated, unstimulated CD4^+^ T cells was determined with flow cytometry. The following antibodies were used: FITC anti-human CD4, APC anti-human CD25, and PE anti-human CD127 (Becton Dickinson). Data were acquired on an LSRII cytometer (Becton Dickinson) and analyzed by using FlowJo software (TreeStar*,* Ashland, OR, USA).

### Plasma cytokines and innate immune function

Plasma from blood drawn in heparin tubes was obtained and stored at −80°C for subsequent quantification of circulating plasma IL-6, IL-8, and IL-10. Innate immune function was assessed with whole-blood *ex vivo* lipopolysaccharide (LPS)-induced tumor necrosis factor (TNF)α production capacity as follows. Within 30 minutes of collection, 50 μl of whole blood was added to previously prepared stimulation tubes and incubated for 4 hours at 37°C. The stimulation tubes contain 0.5 ng/ml LPS (phenol-extracted from *Salmonella abortus equii* (Sigma). After 4 hours, the supernatant was collected and stored at −80°C for subsequent cytokine analysis. Stimulation studies were performed in duplicate, with average values reported. Cytokine production from unstimulated plasma samples and from stimulated whole blood was quantified with chemiluminescence by using the Immulite automated chemiluminometer (Siemens Healthcare Diagnostics, Deerfield, IL, USA). Severe innate immune suppression was defined as an *ex vivo* LPS-induced TNF-α production capacity of less than 250 pg/ml, based on our previous studies of critically ill children [3,4].

### Definitions and statistical analysis

Clinical data were obtained from the electronic medical record. Severity of illness was determined with pediatric risk of mortality (PRISM III), pediatric logistic organ dysfunction (PELOD), and organ-failure index (OFI) scoring within 24 hours of sepsis onset [20-22]. Our primary outcome measure was the occurrence of either persistent infection or new nosocomial infection. For bloodstream and urinary tract infections, persistent infection was defined as consecutive positive blood or urine cultures for greater than or equal to 7 days. For lower respiratory tract infections, persistent infection was defined as consecutive positive endotracheal cultures with ongoing symptoms of pulmonary infection, including purulent secretions (defined as the presence of white blood cells on Gram stain of lower respiratory culture); hypoxia or need for invasive mechanical ventilation; and infiltrate(s) on chest radiographs for greater than or equal to 7 days. For all sites of infection, initial infection was presumed to be resolved if subsequent cultures were negative or if the site was not re-cultured. New nosocomial infection was defined according to CDC criteria and included any new bacterial or fungal infection occurring after 48 hours of sepsis onset [23]. Daily cultures were not obtained as part of the study protocol, although it is routine practice in our PICU to obtain blood, urine, and lower respiratory cultures daily with any fever and to obtain daily blood cultures in patients with bacteremia until two consecutive blood cultures are negative. For patients with community-acquired septic shock, sepsis onset was defined as the time of initial presentation to the emergency department. For patients with nosocomial sepsis as their study-enrollment event, sepsis onset was defined as the time at which the patient met criteria for septic shock. ICU-free days were determined based on physical admission to and discharge from the ICU. Patients who died in the ICU were considered to have zero ICU-free days. Mortality was defined as death during hospitalization.

Comparisons between groups were analyzed with the Mann–Whitney *U* test for continuous variables and the Fisher Exact test for categoric values. Differences in values over time were compared between groups by using analysis of variance. Data are presented as median (interquartile range). A *P* value < 0.05 was considered to be significant throughout. Data were analyzed by using Prism6 software (GraphPad Inc*.,* La Jolla, CA, USA).

## Results

### Subjects

We enrolled 22 sepsis patients and eight healthy children. Healthy subjects had a median age of 12 (3.8 to 14) years (*P* = 0.3 versus sepsis patients), and 50% were girls. Demographic information for sepsis patients is displayed in Table 1. Among sepsis patients, 36% had a complex chronic condition, most commonly developmental delay (*n* = 5). Two patients in the cohort had an oncologic diagnosis. Neither patient was leukopenic at the time of sampling, and neither was receiving chemotherapy at the time of sepsis onset. No transplant patients were in the cohort. Septic shock was community acquired in 20 (91%) cases. Of the 18 patients with positive cultures, seven (39%) had gram-positive infection, four (22%) had gram-negative infection, three (17%) had mixed bacterial infection, two (11%) had viral infection, and two (11%) had mixed viral and bacterial infections. All patients were treated with parenteral antibiotics. All patients with positive growth of bacterial pathogens received correct antibiotic coverage (based on susceptibilities of cultured organisms) with a median time of 2.7 (1.2 to 5.9) hours from sepsis onset to the first dose of parenteral antibiotic.

**Table 1:** Patient demographics

**Characteristic**	**All**	**Persistent or nosocomial infection,**	**No persistent or nosocomial infection**	***P***^**a**^
	**(*n*** **= 22)**	**(*n*** **= 6)**	**(*n*** **= 16)**	
Age, years	1.3 [0.3–14]	8 [0.9 – 16]	0.9 [0.2–12]	0.1
Female gender, *n* (%)	10 (45)	2 (33)	8 (50)	0.6
Complex chronic condition, *n* (%)	8 (36)	3 (50)	5 (31)	1
Initial OFI ≥ 3, *n* (%)^b^	10 (45)	5 (83)	5 (31)	0.06
Initial PRISM III^b^	11.5 [9 – 14]	10.5 [8.8 – 12.5]	13 [8 – 16]	0.4
Initial PELOD^b^	12 [11 – 18]	12 [1 – 14]	12 [11 – 21]	0.3
Site of initial infection, *n* (%)				
Culture negative	4 (18)	0	4 (25)	
Blood	4 (18)	2 (33)	2 (13)	
Lung	8 (36)	3 (50)	5 (31)	
Urine	2 (9)	0	2 (13)	
Abdomen	1 (5)	0	1 (6)	
Multisite	3 (14)	1 (17)	2 (13)	
Glucocorticoid use, *n* (%)^c^				
MP/Dex for < 24 hours^d^	8 (36)	1 (17)	7 (44)	0.4
MP/Dex for > 24 hours	4 (18)	3 (50)	1 (6)	**0.046**
HCTZ for > 24 hours^d^	4 (18)	0	4 (25)	0.5
Outcomes				
ICU-free days in 28 days	17 [10 – 21]	5.5 [0–16]	19.2 [15 – 22]	0.05
Mortality, *n* (%)	2 (9)	1 (17)	1 (6)	0.4

^a^Persistent or nosocomial infection versus no persistent or nosocomial infection (Mann–Whitney or Fisher Exact test). ^b^Values at the time of sepsis onset. ^c^Glucocorticoid use within 7 days of sepsis onset. ^d^Includes two patients treated with HCTZ and with MP/Dex for less than 24 hours. PRISM, Pediatric Risk of Mortality; PELOD, Pediatric Logistic Organ Dysfunction; OFI, Organ Failure Index; MP, methylprednisolone; Dex, dexamethasone; HCTZ, hydrocortisone. Data are median (IQR), except where otherwise specified.

Six (27%) patients developed either persistent infection or new nosocomial infection. No significant differences in clinical characteristics were found between patients who developed persistent or new nosocomial infection and those who did not, with the exception of the use of methylprednisolone or dexamethasone for greater than 24 hours (Table 1). Overall, 64% of patients were treated with glucocorticoids within 7 days of sepsis onset. In the majority of cases, glucocorticoid use consisted of dexamethasone for less than 24 hours, with the intention to prevent or treat postextubation stridor. Of the patients who received methylprednisolone or dexamethasone for greater than 24 hours, two patients were treated with a methylprednisolone infusion for acute respiratory distress syndrome; one patient was treated with methylprednisolone for reactive airways disease; and one patient was treated with dexamethasone in the setting of meningitis.

Characteristics of patients with persistent (*n* = 3) or new nosocomial (*n* = 3) infection are listed in Table 2. Of the patients with persistent infection, one patient had persistent pneumonia for 14 days. The other two patients had persistent bacteremia for 7 and 9 days, respectively. The three nosocomial infections were diagnosed within 1 month of sepsis onset, with one infection occurring in the first week of illness (Day 5) and the other two occurring later (Day 25 and Day 27).

**Table 2:** Characteristics of sepsis patients with persistent or nosocomial infection

**Persistent or nosocomial**	**Age (years)**	**Complex chronic condition**	**PRISM**^**a **^**III**	**OFI**	**Organism**	**Site**
Persistent	9	Metastatic dysgerminoma	14	3	*Pseudomonas*	Lung
Persistent	6	None	12	4	MRSA	Blood
Persistent	16	None	10	3	MRSA	Blood, Lung
Nosocomial	0.8	None	5	2	*Pseudomonas*	Blood, Lung
Nosocomial	16	SGS, TPN dependence	10	3	*Escherichia coli Pseudomonas*	Blood
Nosocomial	0.9	Chromosomal abnormality, developmental delay	11	3	*Enterobacter*	Lung

^a^At the time of sepsis onset. PRISM, Pediatric risk of mortality; OFI, organ failure index; SGS, short-gut syndrome; TPN, total parenteral nutrition.

### Systemic cytokines

As expected, for all sepsis patients on days 1 to 2 of illness, plasma levels of proinflammatory cytokines, IL-6 and IL-8, were significantly elevated compared with healthy controls (82 (21 to 396) pg/ml versus < 6 pg/ml and 50 (19 to 92) pg/ml versus < 15 pg/ml, respectively). Among sepsis patients, early plasma proinflammatory cytokine levels tended to be higher in patients who developed persistent or nosocomial infection compared with those who did not, although this difference did not reach statistical significance. Early plasma levels of the antiinflammatory cytokine, IL-10, were significantly higher in patients who went on to develop persistent or nosocomial infection (Table 3).

**Table 3:** Plasma cytokine levels within 48 hours of septic-shock onset

**Cytokine**	**Persistent or nosocomial infection,**	**No persistent or nosocomial infection,**	**Healthy controls**	***P***
	**(*n*** **= 6)**	**(*n*** **= 16)**	**(*n*** **= 8)**	
IL-6 pg/ml	265 (35–792)	82 (15–396)	< 6	0.3
IL-8 pg/ml	77 (48–176	42 (17–74)	< 15	0.1
IL-10 pg/ml	36 (13–64)	10 (10–29)	10 (10 – 13.9)	**0.04**

*P* values are for persistent or nosocomial infection versus no persistent or nosocomial infection (Mann–Whitney). Data are expressed as median (IQR).

### Adaptive immune response

Despite the presence of a systemic inflammatory response and high levels of circulating cytokines, CD4^+^ T cells isolated from sepsis patients within 48 hours of sepsis onset demonstrated significantly reduced ability to produce the proinflammatory cytokine, IFN-γ, compared with healthy controls (74 (27 to 146) pg/ml versus 606 (169 to 1,169), *P* = 0.002). Early T-cell cytokine-production capacities of the antiinflammatory cytokines, IL-4 and IL-10, were also lower in sepsis patients as a whole compared with healthy controls (2.9 (0.7 to 5) versus 8.2 (3 to 10) pg/ml; *P* = 0.03; and 23 (6 to 41) versus 68 (26 to 126) pg/ml, *P* = 0.02), respectively.

**Figure 1:**
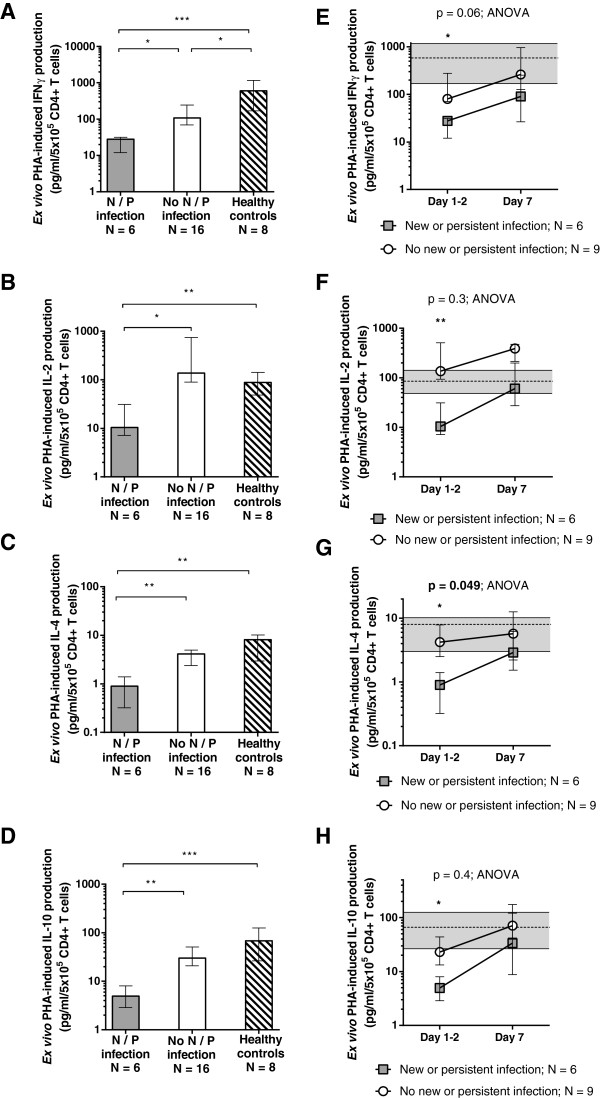
**Adaptive immune response.** Adaptive immune function measured by *ex vivo* PHA-induced cytokine-production capacities within 48 hours of sepsis onset **(A-D)** were lower for children who went on to develop persistent or nosocomial infection (gray bars) compared with sepsis children who recovered without infectious complication (white bars) and healthy controls (striped bars). For those children with day 7 data available **(E-H)**, *ex vivo* PHA-induced cytokine-production capacities for both groups tended to improve over time. Bars **(A-D)** and symbols **(E-H)** represent median values, with error bars representing interquartile range throughout. Dashed lines and shaded areas **(E-H)** represent median and interquartile ranges for healthy control subjects. (*P < 0.05; **P < 0.01; ***P < 0.001).

Among sepsis patients, the development of persistent or new nosocomial infection was associated with lower Day 1 to 2 CD4^+^ T-cell cytokine-production capacities (Figure 1A-D). This was apparent for each cytokine evaluated, including the proinflammatory cytokines (IFN-γ and IL-2) and antiinflammatory cytokines (IL-10 and IL-4). For those patients with Day 7 samples available (*n* = 15), adaptive immune function tended to improve over time in both groups (Figure 1E-H).

### Innate immune response

**Figure 2:**
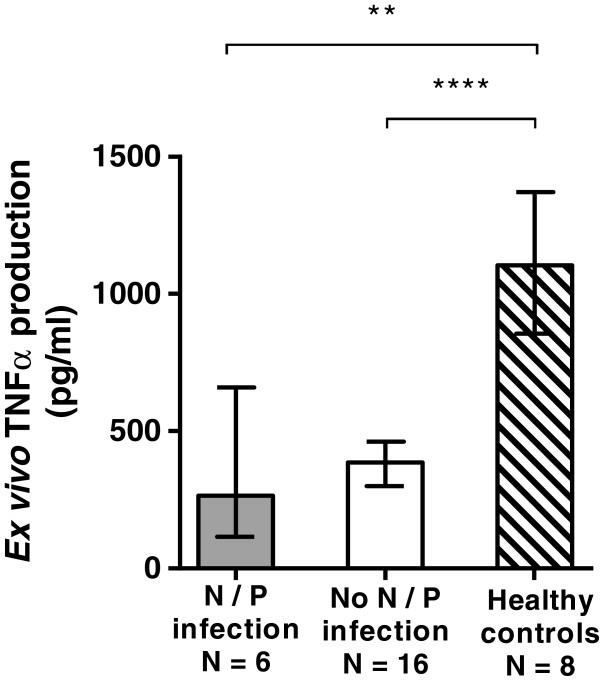
**Innate immune response.** Innate immune function measured by *ex vivo* LPS-induced cytokine-production capacities within 48 hours of sepsis onset were lower for sepsis patients compared with healthy controls. Differences were not seen in early innate immune function between sepsis children who developed persistent or nosocomial infection and sepsis children who did not. Bars and error bars represent median values and interquartile range (**P < 0.01; ****P < 0.0001).

Similar to the adaptive immune response, sepsis patients demonstrated significantly reduced innate immune function on Days 1 to 2 of illness compared with healthy controls (Figure 2). Early innate immune function did not differ between those who went on to develop persistent or nosocomial infection and those who did not (Figure 2). However, only two patients in the cohort demonstrated persistent, severe innate immune suppression (*ex vivo* TNF-α production capacity < 250 pg/ml on Day 7). Both of these patients died during their hospitalization compared with zero deaths among patients with Day 7 *ex vivo* TNF-α production capacity > 250 pg/ml (*P* = 0.008; OR, 145; 95% CI, 2.3 to 9,146).

### Absolute lymphocyte count and regulatory T cells

**Figure 3:**
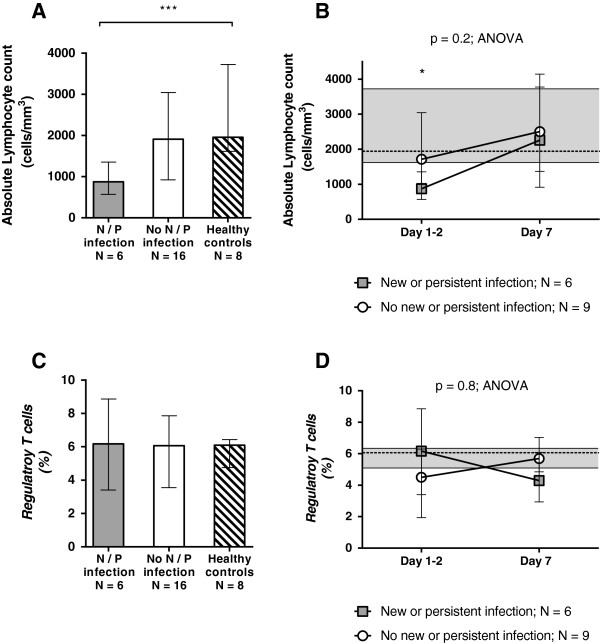
**Absolute lymphocyte counts and regulatory T cells.** Sepsis patients who went on to develop persistent or nosocomial infection (gray bars) had lower absolute lymphocyte counts within 48 hours of sepsis onset **(A)** but not on day 7 of illness **(B)** compared with sepsis patients who recovered without infectious complication and healthy controls **(A)**. No differences were seen among groups in percentage of regulatory T cells measured within 48 hours of sepsis onset **(C)** or over time **(D)**. Bars **(A, C)** and symbols **(B, D)** represent median values, with error bars representing interquartile range throughout. Dashed lines and shaded areas **(B, D)** represent median and interquartile ranges for healthy control subjects (*P < 0.05; ***P < 0.001).

To evaluate further the adaptive immune suppression in sepsis patients, we determined absolute lymphocyte counts and percentages of Treg. Absolute lymphocyte counts tended to be lower in sepsis patients compared with healthy controls. They were lowest for sepsis patients who developed persistent or new nosocomial infection (Figure 3A, B). Regarding Treg in sepsis patients, the percentage of Treg was not different between groups on Days 1 to 2 or on Day 7 (for the smaller cohort of children with Day 7 data available). Furthermore, sepsis children overall had a similar percentage of Treg compared with healthy controls (Figure 3C, D).

## Discussion

In this cohort of critically ill children with septic shock, early adaptive immune suppression, as measured by reduced CD4^+^ T-cell cytokine-production capacity was associated with the development of persistent or nosocomial infection. As cell numbers were controlled for in our experimental model, this suggests that decreased cellular responsiveness in addition to lymphopenia may be associated with adverse infection-related outcomes in this cohort.

The well-recognized signs and symptoms of sepsis are manifestations of an overwhelming inflammatory response. However, it is increasingly recognized that counterregulatory mediators that impair immune cell responsiveness are released shortly after (or at the same time as) proinflammatory mediators [5,7-9,11,24,25]. This results in a state of immune suppression, which is an increasingly recognized sequela of critical illness. In critically ill adults with sepsis, alterations of both innate and adaptive immune function, including elevated circulating levels of proinflammatory cytokines, decreased monocyte MHC class II (for example, HLA-DR) expression, and lymphocyte apoptosis have all been associated with poor outcomes, suggesting that dysregulated immune responses may be important contributors to morbidity and mortality in the ICU [7,10,11,26].

In critically ill children, innate immune suppression has been well characterized across a variety of diagnoses [3-6,27]. In previous studies, the inability of innate immune cells to produce proinflammatory cytokines in response to stimulation has been independently associated with increased risks of mortality and nosocomial infection in critically ill children with influenza, multiple organ dysfunction, respiratory syncytial virus infection, or after cardiopulmonary bypass [3-6].

Data regarding the adequacy of the adaptive immune response in critically ill children are fewer. Two previous studies evaluated absolute lymphocyte counts in critically ill children. In one study of 113 critically ill children, an absolute lymphocyte count of < 1,000 cells/mm^3^ for at least 3 days was associated with the development of nosocomial infection. Meanwhile, prolonged lymphopenia (ALC < 1,000 for longer than 1 week) was independently associated with the development of nosocomial infection, multiple organ dysfunction, and mortality [28]. In a subsequent single-center study of 24 children with sepsis-induced multiple organ dysfunction syndrome, nonsurvivors demonstrated lower absolute lymphocyte counts compared with survivors as early as 3 days after the onset of MODS, although earlier time points were not evaluated in this study [29]. In a multi-center study, Wong *et al.*[18] evaluated genomic expression patterns in 84 critically ill children with systemic inflammatory response syndrome, sepsis, or septic shock, and demonstrated significant downregulation of genes related to antigen presentation and T-cell function in septic shock patients as early as the first day of illness [18]. Subsequent studies similarly demonstrated downregulated expression of genes involved in adaptive immunity in children with septic shock [30,31]. Thus far, these analyses have been limited to genomic-expression profiles and have not directly evaluated lymphocyte function. Ours is the first study to quantitate lymphocyte cytokine-production capacity as a measure of adaptive immune function in critically ill children. In our study, *ex vivo* PHA-induced production of both pro- and antiinflammatory cytokines from isolated CD4^+^ T cells was decreased in sepsis patients compared with healthy controls and was lowest in children with sepsis who developed persistent or nosocomial infection. These decreases were most apparent early in the course of illness (within the first 48 hours of sepsis onset).

Temporal relations between innate and adaptive immunity are likely important. Previous adult studies suggest that adaptive immune suppression in the setting of sepsis may lag behind earlier alterations in innate immune function [10,14]. In our study, we found much earlier evidence of adaptive immune suppression, with the degree of suppression being related to the risk of developing infectious complications. Whether this may suggest a physiologic difference between adults and children or may reflect differences in timing of presentation relative to the onset of septic disease is unclear. It is notable that our work is in agreement with the aforementioned genomic-based analyses of children with sepsis, which found alterations in gene expression related to adaptive immunity evident on the first day of illness [18].

Although early decreases were also seen in innate immune function in our cohort, innate immune suppression was not related to the development of persistent or nosocomial infection. This is likely because the vast majority of children recovered innate immune function within 7 days. Indeed, in keeping with previous studies, the only two children in the cohort with persistent severe innate immune suppression were the only two mortalities in the cohort. Unfortunately, because of the small sample size and few patients with persistent immune suppression, we are unable to comment further on relationships between innate and adaptive immune function over time. We view this as an important avenue of future research.

Mechanisms of the observed adaptive immune suppression are unclear. Lymphopenia and lymphocyte apoptosis are well recognized in septic disease [9,28,32]. In our study, patients who went on to develop new or persistent infection also demonstrated decreased absolute lymphocyte counts early in their illness. Although we did not directly measure lymphocyte apoptosis in this study, it is notable that differences in cytokine-production capacity were apparent despite using equal numbers of lymphocytes in our stimulation assays. This suggests that factors in addition to lymphocyte cell death alone may contribute to adaptive immune suppression in children with sepsis.

Treg are a subset of CD4^+^ T cells with immunosuppressive function. Previous studies have implicated Treg in sepsis-induced adaptive immune suppression in adults [10,13,14,33]. More recent studies, however, have questioned the importance of Treg in the setting of sepsis [15,34]. In agreement with these recent studies, our study also failed to show significant differences in percentage of Treg in children with sepsis compared with healthy controls or in children who developed persistent or nosocomial infection compared with those who did not. Likewise, we did not see evidence for TH_2_ skewing by cytokine-production profile, but rather cytokine-production capacity was reduced across the board.

For some patients in the cohort, glucocorticoid use may have contributed to adaptive immune suppression. Interestingly, hydrocortisone use was not associated with the development of persistent or nosocomial infection in our cohort. Although the debate continues over hydrocortisone use in children with septic shock, it is important to consider that more powerful antiinflammatory glucocorticoids (for example, dexamethasone or methylprednisolone) may be used in sepsis patients to manage acute comorbidities such as reactive airways disease and acute respiratory distress syndrome. Given the presence of adaptive immune suppression in the setting of sepsis and the association between antiinflammatory glucocorticoids and adverse outcomes in our cohort, we believe this is an important area for future prospective study.

Our study has important limitations, including a small sample size. Thus, we cannot perform multivariable analyses to account for other baseline differences between groups that could have confounded our results. Our study is also limited by the fact that many patients recovered before day 7 sampling. It is possible that we might have detected greater differences between groups in both innate and adaptive immune function in the subacute phase if day 7 samples were available for all patients. Likewise, with only two sampling windows, we are unable to determine patterns of immune function at other points in time. Last, as we chose to compare sepsis children with healthy controls, we cannot comment on the specificity of our findings to septic shock. Adaptive immune suppression may also occur in other critically ill pediatric populations, which we view as an important area of future study.

## Conclusions

This cohort of critically ill children with septic shock demonstrated early adaptive immune suppression compared with healthy children, with further suppression seen in children who went on to develop infectious complications. This was characterized by lower CD4^+^ T-cell cytokine-production capacities and lymphopenia, but not by increased percentage of regulatory T cells. Future studies are needed to confirm these findings in a larger cohort of patients, to investigate potential mechanisms of adaptive immune suppression in pediatric sepsis, and to understand the interplay between innate and adaptive immune responses in children with sepsis.

## Key messages

• CD4^+^ T cells isolated from children with septic shock show decreased ability to produce both pro- and antiinflammatory cytokines in response to *ex vivo* stimulation.

• Among children with sepsis, suppressed CD4^+^ T-cell cytokine-production capacity is associated with the development of persistent or new nosocomial infection.

## Abbreviations

IFN: Interferon; IL: interleukin; LPS: lipopolysaccharide; OFI: Organ Failure Index; PELOD: pediatric logistic organ dysfunction; PHA: phytohemagglutinin; PRISM: pediatric risk of mortality; TNF: tumor necrosis factor; Treg: Regulatory T cell.

## Competing interests

The authors disclose no financial or nonfinancial competing interests.

## Authors’ contributions

JM designed the study, screened and enrolled patients, performed experiments, analyzed data, and drafted the manuscript. RN screened and enrolled patients, performed experiments, analyzed data, and revised the manuscript. KG and LS screened and enrolled patients, collected and managed data, and revised the manuscript. LH and JN processed patient samples, performed experiments, and revised the manuscript. MH designed and supervised the study and drafted the manuscript. All authors read and approved the final manuscript.
